# Book Notices

**Published:** 1855-05

**Authors:** 


### BOOK NOTICES.

*Cases of Polypus of the Womb.* By WALTER CHANNING, M.D.

*Lectures in Reply to the Croonian Lectures for* 1854, of CHARLES WEST, M.D., London. By HENRY MILLER, M.D., Professor of Obstetric Medicine in the University of Louisville, &c. &c.

*Oration before the Physico-Medical Society of New Orleans.* By A. MERCIER, M.D., &c.

*Dr. John H. Griscom's Anniversary Discourse for* 1854.

*Report of the Pennsylvania Hospital for the Insane, for the Year* 1854. By THOMAS S. KIRKBRIDE, M.D., Physician to the Institution

*Twelfth Annual Report of the Managers of the State Lunatic Asylum, N. Y.*

Dr. CHANNING has reported thirteen cases of uterine polypus which has come under his own observation. The pamphlet is republished from the Boston Medical and Surgical Journal.

Dr. Miller's Lectures are devoted to the defence of the speculum, and topical medication of the os uteri against the attacks of Dr. West. We have not space for an analysis even of the lectures, but commend them to the attention of those interested in the controversy. It may be that the true value of the speculum as a means of diagnosis has not yet been determined. We have no doubt but that it has been abused, but in our own practice it has revealed to us facts in regard to the pathology of the os and corvix uteri, that we did not know how to acquire in any other manner.

A notice of the lectures of Dr. West will be found on another page.

Dr. Mercier's discourse is upon the progress of surgery since the commencement of the present century. Among the improvements due to American surgeons, Dr. M. records the extirpation of the ovary practised in this country by Drs. McDowel and Nathan, and Alban Smith, before it was advocated by Morand and Lizars. The two inferior thirds of the sternum and the extremity of the corresponding ribs were resected in the surgical wards of the Charity Hospital in New Orleans in 1846. Dr. Wedderburn removed the radius and cubitus entire in 1852. Dr. W. H. Deaderick of Rogersville, Tenn., removed a portion of the inferior maxillary bone in 1810, two years before Dupuytran performed the same operation, which added so much to his reputation. Dr. Mott, in 1813, first passed a ligature around the brachiocephalic trunk and in 1827 first ligated the common iliac. This operation was afterwards performed by Crampton, of Dublin, without success, and by C. A. Luzenberg, of New Orleans, resulting in a perfect cure of his patient. Dr. Luzenberg also twice extirpated the parotid gland.

Dr. Mercier does not allude to the triumphs of Dr. Dudley in the operation for stone.

Among those aids which America has rendered to progressive surgery the discovery of ether and chloroform, and their application for the production of anæsthesia, is considered by our author—and very justly, we think—the most prominent.

Accompanying the lecture is the report of a successful operation for ovariotomy.

The Anniversary Discourse of Dr. Griscom before the New York Academy of Medicine is devoted to a discussion of "the relation between the people and the science of medicine." It is well written, and calculated to interest both physician and people.

The Report of the Pennsylvanian Hospital for the Insane contains, in addition to the usual tables, an eloquent tribute to the memory of the late Jacob G. Morris, one of the managers of the institution and who perished on the ill-fated Arctic.

The Report of the Managers of the New York State Lunatic Asylum coontains much that is interesting to physicians.

Of 360 patients, the disease was hereditary in 142, or 29-97 per cent of the whole number admitted, and 36-41 per cent had insane relatives. J.

---

*An Inquiry into the Pathological Importance of the Ulceration of the Os Uteri:* beeing the Croonian Lectures or the Year 1854. By CHARLES WEST, M.D., &c. &c. Philadelphia: Blanchard and Lea.

THE views of Dr. West on this *questio-vexata* must be regarded with interest by the prefession. We have therefore read with care these lectures, with the hope of finding some new light thrown upon the pathology and significance of uterine ulceration.

After describing the changes through which the uterus passes during pregnancy, and after parturition, and some of the morbid conditions sometimes dependent, partially at least, on those changes, and at others independent of them, the question is asked how are they explained? Do they proceed from an invariable pathological occurrence, which is present in every case, how wide soever may be in other respects the points of difference, or are they the indications of disordered function, which may depend on causes various as those which produce vomiting or occasion dyspnœa?" This inquiry constitutes the subject of the lectures, and if we understand it aright, it is this. Is ulceration of the mouth of the womb a local disease, producing the symptoms usually accompanying it? or is it itself one of a numerous train of symptoms indicating functional derangements, dependent upon various causes? The author maintains the latter proposition.

Dr. W· gives the following descriptton of ulcerations, or, as he terms them, abrasions of the os uteri:

The ulcerations to which such important results are attributed are, for the most part, mere superficial abrasions of the epithelium investing the lips of the os uteri, whose abraded surface is of a vivid red color, and finely granular. In other cases, in which the absence of epithelium is less complete, the surface seems beset by a large number of minute, superficial, apthous ulcerations, between which the tissue appears healthy, or slightly redder than natural. The ulcerations of the os uteri seldom or never present an excavated appearance, with raised edges, as ulcers of other parts often do; but either their surface is smooth, or it projects a little beyond the level of the adjacent tissue. They are usually, but not constantly, of greater extent on the posterior than on the anterior lip, are sometimes confined to the former, but very rarely indeed limited to the latter. They appear to commence at the inner margin of the os uteri, whence they extend onwards, and sometimes, though by no means invariably, the short extent of the canal of the cervix uteri which can be brought into view by the speculum, appears denuded of its epitheliu. The adjacent parts of the os uteri vary considerably in their appearance: sometimes their natural pale rose tint is preserved up to the abrasion or edge of the abrasion, which is marked by a distinct, well-defined line, while at other times the whole surface is of a much more vivid red than natural, and the line of demarcation between the abraded and the healthy surface is irregular and indistinct. the one encroaching on the other. The orifice of the uterus is generally more open than in a state of health; and the disappearance of the abrasion, which always takes place from the periphery towards the centre, is accompanied by the gradual closure of the previously patent orifice. The state of the ti sue of the os and cervix varies; sometimes there is a very marked softness of the parts, the condition resembling that of the uterus soon after abortion or delivery, while at other times it is much harder than natural; but it certainly is not at all a common occurrence for extensive abrasion of the os uteri to coexist with a condition of the organ such as would seem healthy to the touch. The secretion from the surface varies considerably in different cases, and the chief part of the leucorrhœal discharge from which patients suffer is derived from withn the canal of the cervix, or from the cavity of the womb—not from the abrasion itself. Still, in some instances, those especially in which the ulceration presents a very marked granular character, the discharge derived from this source alone is far from inconsiderable. The degree of sensibility which the ulcerated surface possesses also varies greatly; now and then the slightest touch is extremely painful; but in the majority of cases, the ulcerated surface is not more sensitive than the adjacent parts. nor is the neck of the uterus, whose os is abraded, by any means constantly more tender to the touch than the same part of an organ entirely free from that infection.

From the analysis of a large number of cases tabulated in Lecture Second, the following conclusions are derived:

1st. Uterine pain, menstrual disorder, and leucorrhœal discharges—the symptoms ordinarily attributed to ulcernation of the os uteri—are met with independently of that condition almost as often as in connection with it.

2d. These symptoms are observed in both classes of cases with a vastly preponderating frequency at the time of the greatest vigor of the sexual functions, and no cause has so great a share in their production as the different incidents connected with the active exercise of the reproductive powers. But it does not appear that ulceration of the os uteri exerts any special influence, either in causing sterility or in inducing abortion.

3d. While the symptoms are identical in character in the two classes of cases, they seem to present a slightly increased degree of intensity in those instances in which ulceration of the os uteri existed.

4th. In as far as could be ascertained by careful examination, four-fifths of the cases of either class presented appreciable changes in the condition of the uterus—such as misplacement, enlargement, and hardening of its tissue, while frequently several of these conditions co-existed. An indurated or hypertrophied state of the cervix uteri was, however, more frequent in connection with ulceration of the os uteri than independent of that condition.

5th. The inference, however, to which the last-mentioned fact would seem to lead, as to the existence of some necessary relation, such as that of cause and effect—between ulceration of the os uteri and induration of its cervix, is in great measure negatived by two circumstances :—

1. The number of instances in which an indurated cervix coexisted with a healthy os uteri.

2. The fact that, while induration of the cervix was present in twenty-five out of forty-six cases, in which the ulceration of the os uteri was very slight, it was altogether absent in nine out of sixteen cases in which the ulceration was noted as having been very extensive.

In the third lecture, Dr. West enters into a consideration of the causes of uterine disorder. Numerous cases are reported, tending to illustrate and sustain the proposition contained in the following short extract:

Unfortunately, I can put forward no such pretensions, for I believe that instead of the different symptoms which are supposed to depend on ulceration of the os uteri being produced by that or by any other single cause, they in reality arise from very various causes; that at one time they attend on general constitutional disorder, at another on some ailment of the sexual system, and that ailment by no means the same in every instance. If this be so, however; instead of the consideration of one pathological condition of the uterus and its possible consequences, we have to inquire into little less than uterine disorders in general, their causes and their symptoms—an undertaking which would occupy not one lecture, but several; and the preparation for which would be the study of a lifetime.

In this controversy Dr. West seems to us to state the proposition of his opponents in an unfair light. The most strenuous advocates of topical medication of the os and cervix uteri admit that in many instances the symptoms that accompany ulceration of those tissues may be dependent on other causes, and that for their proper treatment, not only is a knowledge of the whole catalogue of uterine diseases necessary, but that, as an aid in diagnosis and an adjuvant to other remedies, the speculum and topical agents are invaluable. Dr. W. himself admits that local medication is sometimes necessary, especially in that condition of the mucous membrane known as granular metretis. For our own part, we have adopted the maxim: *in medias res ibis tuitssimus.* We are no advocate for specialities narrowed down to the treatment of a single affection, whatever it may be. Devotion to a single organ, or a single disease, necessarily results in injury to both physician and patient, and the man who sees in every pelvic pain his favorite malady is fitly described by our author.

He unlearns what physiology might teach him of the uterus and its functions, and sees in all the varied manifestations of disorder the expression of one fact, and of one alone; namely, the existence of ulceration of the womb, and its reaction first on the uterine system, then on the general health. For him, indeed, there is little more to learn in uterine pathology; for when once a case has been ascertained not to be one of fibrous tumor, polypus, or cancer, then ulceration of the os uteri is the almost invariable cause to which the symptoms are referred, and the cure of this ulceration is the one grand object at which he endeavors. All the evils inseparable from the practice of a speciality are thus aggravated, and the natural tendency of such practice to subside into routine, or to degerate into empiric.sm (I use the word in no invidious sense), becomes almost unavoidable.

After discussing the dangers from the use of the agents ordinarily employed, the Dr. remarks:

At the same time there are some exceptional cases, which I have already referred to, where ulceration, or some allied morbid condition of the os uteri, is found to exist independent of any appreciable disease elsewhere; and others, equally rare, in which, after symptoms of uterine ailment have been subdued, a morbid state of the os uteri persists which is benefited by stimulant applications. In such cases, I use either the nitrate of silver or the acid nitrate of mercury, though neither of them frequently; and for weeks together no case appears among my patients at St. Bartholomew's Hospital in which the employment of either appears to me indicated. J.

---

*A Systematic Treatise, Historical, Etiological and Practical, on the Principal Diseases of the Interior Valley of North America, &c., &c.* By DANIEL DRAKE, M. D. Edited by S. HANBURY SMITH, M. D., and FRANCIS G. SMITH, M. D., Second series Philadelphia: Lippincott, Grambo & Co. 1854.

This second volume of Dr. Drake's great work has been on our table some time, and we should have noticed it earlier but that we wished first to give it a careful examination. This we have not yet been able to do, but from what we have seen of it, we can assure our readers that it will not diminish in the least the reputation of its distinguished author, of whom we in the West may justly feel proud. The two volumes constitute a complete library on the diseases of the great Mississippi Valley.

For sale by A. H. & C. Burley. J.

